# Windscapes shape seabird instantaneous energy costs but adult behavior buffers impact on offspring

**DOI:** 10.1186/s40462-014-0017-2

**Published:** 2014-09-12

**Authors:** Kyle Hamish Elliott, Lorraine S Chivers, Lauren Bessey, Anthony J Gaston, Scott A Hatch, Akiko Kato, Orla Osborne, Yan Ropert-Coudert, John R Speakman, James F Hare

**Affiliations:** 1 Department of Biological Sciences, University of Manitoba, Winnipeg R3T 2N2, Manitoba, Canada; 2 The Buntings, Sandy SG19 2TT, Bedfordshire, UK; 3 Environment Canada, National Wildlife Research Centre, Carleton University, Ottawa K1A 0H3, Ontario, Canada; 4 Institute for Seabird Research and Conservation, Anchorage, AK, USA; 5 Université de Strasbourg, IPHC, 23 rue Becquerel, Strasbourg 67087, France; 6 CNRS, UMR7178, Strasbourg 67087, France; 7 Department of Biology, University of Victoria, Victoria, British Columbia, Canada; 8 Institute of Biological and Environmental Sciences, University of Aberdeen, Aberdeen, UK; 9 State Key Laboratory of Molecular and Developmental Biology, Institute of Genetics and Developmental Biology, Chinese Academy of Sciences, 1 West Beichen Road, Chaoyang, Beijing, CN-100101, PR China

## Abstract

**Background:**

Windscapes affect energy costs for flying animals, but animals can adjust their behavior to accommodate wind-induced energy costs. Theory predicts that flying animals should decrease air speed to compensate for increased tailwind speed and increase air speed to compensate for increased crosswind speed. In addition, animals are expected to vary their foraging effort in time and space to maximize energy efficiency across variable windscapes.

**Results:**

We examined the influence of wind on seabird (thick-billed murre *Uria lomvia* and black-legged kittiwake *Rissa tridactyla*) foraging behavior. Airspeed and mechanical flight costs (dynamic body acceleration and wing beat frequency) increased with headwind speed during commuting flights. As predicted, birds adjusted their airspeed to compensate for crosswinds and to reduce the effect of a headwind, but they could not completely compensate for the latter. As we were able to account for the effect of sampling frequency and wind speed, we accurately estimated commuting flight speed with no wind as 16.6 ms^?1^ (murres) and 10.6 ms^?1^ (kittiwakes). High winds decreased delivery rates of schooling fish (murres), energy (murres) and food (kittiwakes) but did not impact daily energy expenditure or chick growth rates. During high winds, murres switched from feeding their offspring with schooling fish, which required substantial above-water searching, to amphipods, which required less above-water searching.

**Conclusions:**

Adults buffered the adverse effect of high winds on chick growth rates by switching to other food sources during windy days or increasing food delivery rates when weather improved.

## Background

Environmental conditions (e.g. snowpack, obstacles and water or air currents) may shape the energy costs of animal behavior [1]-[5]. Such costs can be reflected in the cost of foraging, such as ducks that must fight against the current to obtain their food [6]-[9]. Alternatively, animals may alter their behavior to minimize those costs, such as caribou that avoid areas of deep snow to reduce locomotory costs [10]. For many animals, energetic costs peak while rearing young [11]-[13] and so to reproduce successfully, foraging must be efficient and adaptable to changing environmental conditions. For flying animals, the windscape is particularly important in determining foraging energetics as it influences flight efficiency and prey accessibility [14]-[19]. However, the effect of windscape on a species depends on its flight style [20]. For instance, high wind speeds reduce foraging costs in petrels that can use wind to soar, but increase foraging costs in auks that are unable to soar and thus must fight against the wind to reach their destination [21],[22].

Not surprisingly, windy weather can reduce adult body mass and chick provisioning rates in some piscivorous birds, ultimately lowering reproductive success [23]-[29]. Adult seabirds, however, are known to buffer variability in food availability so as to maintain constant chick provisioning rates by increasing time spent foraging when food is scarce [30]-[32]. While most studies on buffering by adult seabirds examine how feeding rates vary in response to variation in prey stocks [33]-[35], similar processes likely occur in response to inclement weather. For example, adults may alter time budgets and draw on stored energy reserves to maximize reproductive success by continuously feeding offspring during inclement weather, and recoup those reserves during calmer weather when chick feeding is less costly. Alternatively, given that adult self-feeding (requiring only a single commuting flight) may be less affected by wind than chick provisioning (requiring several commuting trips to bring food back intermittently to the offspring), adults may self-feed when wind speeds are highest and provision chicks at other times. We hypothesize that adults buffer the effects of weather and predict that at-sea behavior would be more strongly correlated with weather than chick growth or feeding rates.

One possible way in which parental birds could alter their behavior to reduce the effect of wind speed is by switching to alternative prey. For instance, schooling fish require more above-water searching (flights and time spent flying; [36],[37]) to locate, especially when wind disturbs the surface layer [13],[14]. In contrast, benthic fish and invertebrates are smaller and/or of lower energy density than schooling fish [38], but are either more abundant (invertebrates) or are associated with bottom features (benthic), which means they require less above-water searching [36],[37],[39],[40]. We predicted that in response to high winds, parental birds would switch to prey items that were more spatially consistent and required less above-water searching.

To understand behavioral responses to wind, it is important to quantify the energy costs associated with variable wind speed. Theoretical flight costs follow a U-shape, increasing at both low and high flight speeds (the power curve; [41]-[43]). Thus, flight costs increase non-linearly with forward flight for birds that are using directed flight (i.e. commuting to a nest site, migrating, flying between food patches), as those birds will presumably be flying in the increasing portion of the power curve. Miniaturized GPS-accelerometers provide a fine-scale estimation of energy costs and flight speeds in wild birds [19],[44],[45]. In the presence of a tailwind that increases ground speed for a given airspeed, the air speed that minimizes energy costs per distance travelled will decrease [43],[46]. Many studies have confirmed that airspeed decreases with tailwind speed in breeding birds [47]-[53], bats [54], migrating dragonflies [55] and migrating birds [56]-[58], and that animals can largely compensate for crosswinds [53],[54].

We studied the impact of wind on thick-billed murres (*Uria lomvia*) and black-legged kittiwakes (*Rissa tridactyla*) at varying temporal scales: seconds (wing beat frequency and flight/swim speed), hours (time spent flying, time spent at colony, energy delivery rate) and days (chick growth rate, daily energy expenditure). These two seabirds are suitable model species for examining the impact of wind on flight because they have relatively high flight costs; murres have the highest flight costs, for their body mass, of any bird [59] and kittiwakes also have high flight costs [60]. We examined multi-scale behavioral responses to wind and predicted that: (i) air speed and energy costs would increase with headwind speed and crosswind speed; (ii) the strength of the correlation between wind speed and behavioral parameters would decrease over increasing temporal scales as adults buffered the effect of wind speed; and, (iii) adults would switch from prey items requiring extensive above-water searching to items require less above-water searching as wind speed increased.

## Methods

All activities were approved under the guidelines of the Canadian Council on Animal Care (protocol F11-020).

### Thick-billed murres

We studied murres at the Coats Island, Nunavut, west colony (62°57?N, 82°00?W) during the chick-rearing period (15 July - 18 August) 1998¿2011. As part of a long-term monitoring study [36],[37],[61]-[63], we collected information on feeding rates, diet, attendance and chick growth rates annually. Starting in 2004, we also collected information on at-sea behavior from time-depth recorders. We found no impact of wind on time spent flying per day (see Results). Thus, in 2006 and 2009, we measured daily energy expenditure using doubly-labelled water to determine whether overall energy costs were impacted. As we found no effect of wind on daily energy expenditure (see Results), we then focused on measures directly associated with flight behavior by attaching GPS loggers and accelerometers in 2010 and 2011 to measure ground speed and wing beat frequency. Throughout, we included only birds with chicks 3¿15 d old because feeding rates are constant for murres with chicks within that age range [63]. To reduce autocorrelation of weather over short time scales, we included data covering many years.

Chick-rearing birds at the Coats Island west colony forage almost exclusively within 100 km to the west of the colony [64]. Therefore, the core foraging area is bounded by the colony and the community of Coral Harbour, roughly 120 km from the colony. We recorded wind speed and wind direction daily (1800 h) at a fixed point near the Coats Island cabin, immediately adjacent to the murre colony, using a handheld anemometer (Davis Industries, Hayward, California). We also downloaded hourly wind speed and wind direction recorded at the Coral Harbour airport (www.weatheroffice.gc.ca). The daily average magnitude of wind in a particular direction at 1800 h was correlated between Coral Harbour and Coats Island camp (R?=?0.58, P?<?0.001). For those variables recorded at the finest time scales, correlations were strongest when weather variables were averaged over the scale of 24 hours, and we therefore used daily averages for the Coral Harbour data (e.g. ground speed: R^2^?=?0.29 over 24 h, R^2^?=?0.24 over 1 h). We used time of day (cos(?*Hours since solar midnight/12)) as a proxy for light levels [65],[66].

### Black-legged kittiwakes

We augmented our more detailed information on murres with information from black-legged kittiwakes collected during chick-rearing (July 2010 and 2012) and incubation (30 May¿16 June 2013) at the radar tower colony on Middleton Island, Alaska (59°27?N, 146°18?W; [67]-[69]). The main benefit of the kittiwake dataset is that because kittiwakes do not dive, we did not need to encase the GPS units in resin and we could use lighter GPS units; we were therefore able to attach both GPS units and accelerometers simultaneously. We endeavoured to collect data on kittiwakes that complemented the data already collected on murres. In 2010, we collected data on time spent flying and feeding rates on chick-rearing birds. In 2010 and 2012 we collected data on chick growth rates. In 2013, we collected GPS-accelerometer data on ten incubating birds. We chose uniformly heavy birds (485?±?20 g) to avoid the confounding effect of body mass on measurements of acceleration and wing beat frequency and to minimize any device effects (see next sections).

As there was a weather station associated with the Middleton Island Airport, and kittiwakes forage closer to the colony than murres, we used wind speed and direction recorded within 1 km of the colony at 20 minute intervals (http://cdo.ncdc.noaa.gov/qclcd/QCLCD). Finally, we also used circularly-transformed time of day (AKDT) as a proxy for light levels at Middleton Island.

### Murre foraging behavior

We used chick growth rates as a proxy for fitness as chick growth rates are correlated with offspring recruitment rates (U. Steiner and A. J. Gaston, unpubl. data); virtually all chicks that hatch ultimately fledge, so fledging success is a poor indicator of fitness at our study site. Chick growth rates, however, link adult foraging behavior to the probability that a chick will recruit to the colony, which is a component of fitness. We recorded chick mass every two to three days for a subset of 25¿50 chicks that were individually marked with metal bands [62]. We completed at least three continuous 24-h or 48-h feeding watches during each season (44 total watches). During the watches, we estimated visually the species and length of all fish delivered to offspring at ~30 breeding sites and used species-specific relationships between total energy and fish length to determine energy delivery rates [38],[61],[63]. We correlated average energy delivery rates per day (total and for each prey type) between 0600 h and 1000 h against wind speed. We were particularly interested in whether feeding rates of schooling fish, which require more above-water searching (flights and time spent flying) to locate, were impacted by wind. We used previously reported data for daily energy expenditure measured via doubly-labelled water [12],[45],[59].

We attached time-depth temperature recorders (Lotek, St John¿s, Canada)¿5-g LTD1100 (sampling interval?=?3 s; N?=?140) in 2004¿2007, 5-g LAT1400 (interval?=?15 s; N?=?20) in 2008 and 3-g LAT1500 (interval?=?15 s; 2009¿2011, N?=?50) in 2009-2011¿to the legs of parental murres and extracted time budgets (time spent flying, resting on water and resting at the colony) from the temperature log [64],[65]. We also attached 17-g M190-D2GT biaxial recorders (sampling rate?=?32 Hz; Little Leonardo, Tokyo, Japan; see [45]) in 2010 (N?=?42) and 2011 (N?=?24), as well as 25-g GPS devices (interval?=?15 s when ground speed above 2.8 m/s; CatTraQ¿, Catnip Technologies, USA) in 2010 (N?=?18) and 2011 (N?=?20) to the back of parental murres using Tesa tape. Whereas the leg-mounted recorders weighing ~0.5% of body weight do not impact murre feeding rates [65], the back-mounted recorders weighing ~1.8-3% of body weight reduced murre feeding rates [64] and imposed increased energy expenditure during flight [12] and reduced dive duration [70]. Because all birds were equipped similarly, we assume that all birds were similarly impacted by the devices.

### Kittiwake foraging behavior

As for murres, we used chick growth rates as a proxy for fitness in that chick growth rates are correlated with offspring recruitment rates at our study site [71]. We recorded chick mass every five days at roughly 40 nests on the radar tower colony [69],[71]. Kittiwakes sometimes raise two chicks (we excluded any nests with three or more chicks), and we calculated chick growth rates separately for single, A-chicks and B-chicks [71]. As we only analyzed residuals (see Statistical Analyses section), we combined residuals from all three groups for analyses. We attached LTD1100 temperature recorders (same as previous section) to the legs of 30 kittiwakes for 48 h and videotaped each site from outside the tower. We used those data to calculate time spent flying, time spent on the water and time spent at the colony [72]. We also attached both 3-g Axy accelerometers (sampling rate?=?50 Hz; Technosmart, Rome, Italy) and 14-g GPS devices (interval?=?30 s; CatTraQ¿, Catnip Technologies, USA) in 2013 (N?=?20) to the back of parental kittiwakes using Tesa tape. The back-mounted GPS-accelerometers weighed ~3.5% of body weight, which is known to impact behavior in other bird species [73]-[75]. We assume that all birds were similarly impacted by the devices because all were equipped similarly.

In 2010, we also injected 0.5 mL of doubly-labelled water into the brood patch and obtained background, equilibrium (1 h of captivity) and final (48 h) blood samples from the brachial vein to measure daily energy expenditure in 37 birds [45],[59],[76]. Samples were timed as close to multiples of 24 h as possible to avoid circadian effects [77]. All samples were run blind to the identity of the bird and converted to values of daily energy expenditure using a single pool model with a fixed 25% evaporative water flux (equation 7?·?17: [78]) and a respiratory quotient of 0?·?81 based on nutrient content of the diet (80% protein, 15% fat, 5% carbohydrate). Using this equation, estimates for energy expenditure based on doubly-labelled water in charadriiform birds are accurate within 2¿18% relative to respirometry values from the same individual [79].

### Accuracy of ground speed measurements

To examine the accuracy of ground speed recorded at 15 s (murres) and 30 s (kittiwakes) intervals, we subsampled commuting (incoming/outgoing) flight tracks at longer time intervals. We recorded ground speed for longer intervals as a proportion of ground speed at 15 s intervals and fitted exponential models to the relationship between step interval and ground speed as a proportion of ground speed at 15 s. We also recorded the instantaneous ground speed as reported by the GPS logger.

**Figure 1:**
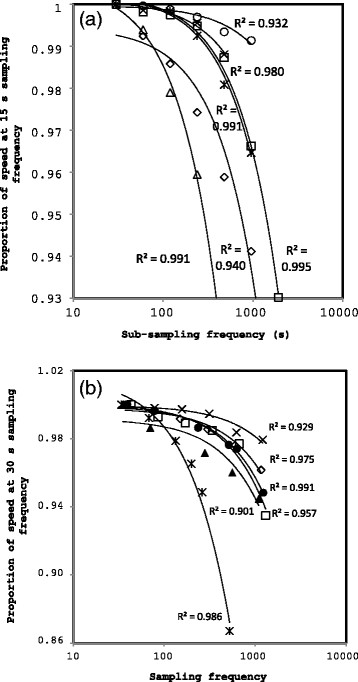
**Average measured ground speed as a proportion of the minimum sampling frequency declines with sub-sampling frequency for (a) murres and (b) kittiwakes, after accounting for random error associated with GPS signal.** Each symbol represents one of six birds chosen randomly from the dataset. Least-squares exponential functions are shown.

Apparent (measured) ground speed decreased exponentially with step size (Figure 1). The average coefficient for the exponential function across all six individuals was 1.00087?±?0.00014 (murres) and 1.00093?±?0.003 (kittiwakes), revealing that at time?=?0 the estimated instantaneous ground speed would be 0.087% higher than the ground speed recorded at 15 s intervals (murres) or 0.093% higher than the ground speed recorded at 30 s intervals (kittiwakes), even after accounting for random error associated with GPS measurements based on location error distribution of stationary GPS units. Thus, recording ground speed at 15 s to 30 s intervals accurately estimated ground speed with <0.1% average error. In contrast, the ¿instantaneous¿ onboard GPS speed readings averaged 0.86?±?0.15 of the value measured at 15 s intervals (murres) and 0.96?±?0.02 of the value measured at 30 s intervals (kittiwakes). Given that the step interval-ground speed graph showed a clear asymptote and that the instantaneous ground speed was *lower* than the ground speed estimated at 15 s increments, we concluded that the instantaneous ¿on-board¿ ground speed was less accurate than the 15 or 30 s estimates. The ¿on-board¿ flight direction also seemed more variable than the GPS tracks would suggest.

These results contradict those of Safi et al. [53], who recommended the use of the instantaneous ¿on-board¿ GPS recordings, although they only compared their values to those obtained at 15 minute intervals and not necessarily for commuting flights; at 15 minutes, ground speed could be substantially underestimated within our dataset (Figure 1). Our step interval-ground speed showed a clear asymptote. In contrast, fractal movement, indicative of fine-scale searching, and which would imply that ground speed itself was dependent on scale, would be expected to have a linear relationship between step size and speed [80]. Thus, our relationship is applicable only to direct commuting flights and not to more convoluted searching behavior.

### Statistical analyses

We completed all statistical analyses in R 2.14.2 (R Core Team 2013). We were interested in the response of behavioral metrics to wind speed after accounting for potential confounding variables. Therefore, for each metric used as a dependent variable we constructed a general linear mixed model with individual as a random factor using time of day (circularly-transformed), year (for parameters measured in multiple years) and wind speed in the direction of travel and wind speed perpendicular to the direction of travel as independent variables. To remove the potential for spurious correlations with wind speed due to potential correlations with date for variables at long time scales, we reported wind speed as the measured wind speed for that date ¿ average wind speed for that date across all years for comparisons with parameters that are expected to vary with date: adult provisioning behavior, chick growth rate, daily energy expenditure and flight time. We included only wind speed and calendar date as independent variables for chick growth rate and daily energy expenditure, as those variables were calculated over >24 h periods.

We calculated the chick growth rate as the difference in body mass between subsequent measurements [81]. We calculated the average population-wide chick growth rate by fitting a linear equation to the average chick mass for a given age across all years. We then calculated the growth rate as the residual for any particular individual for any particular age [81].

For flight speed analyses, we converted all GPS latitudes and longitudes to UTM coordinates (UN_t_?=?UTM Northing at time t; UE_t_?=?UTM Easting at time t) and calculated the average ground speed at time t:

(1) $$\mathrm{Speed}={\left({\left({\mathrm{UN}}_{t+1}¿\;{\mathrm{UN}}_{t}\right)}^{2}+{\left({\mathrm{UE}}_{t+1}¿\;{\mathrm{UE}}_{t}\right)}^{2}\right)}^{0.5}/\left(t_{t+1}?t_{t}\right)$$

For murres, the ground speed distribution was strongly bimodal with a minimum (<0.1%) of measurements at 5 m/s and thus we considered a flight to occur when there were six consecutive measurements?>?5 m/s. For kittiwakes, we used the accelerometer profiles to determine the start and end of flights. We removed the first and last GPS measurement for each flight (which may be biased by the takeoff or landing) and calculated average ground speed for each flight.

**Figure 2:**
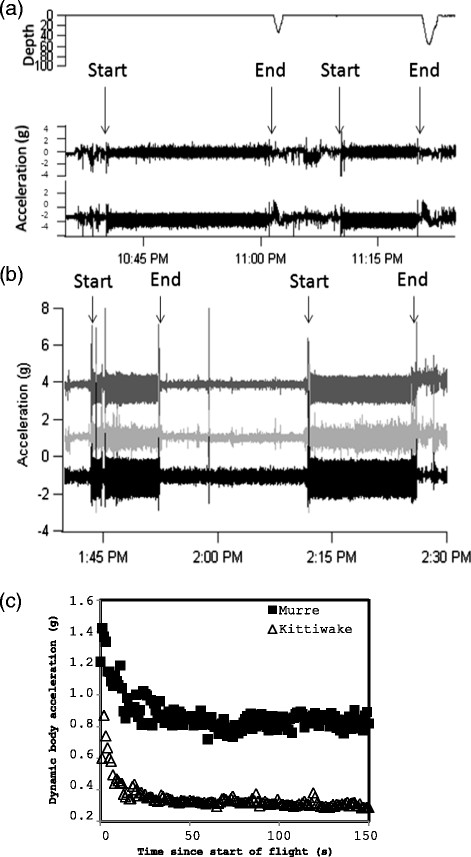
**Typical (a) murre and (b) kittiwake traces from accelerometers.** Both show two flights with the typical increase in acceleration at the start of each flight. For murres, depth is also shown. **(c)** Dynamic body acceleration as a function of time since the start of the flight (N = 10 individuals for each species).

**Figure 3:**
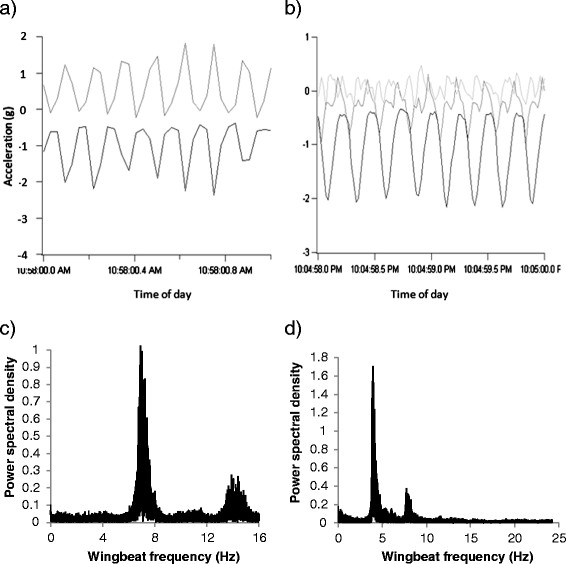
**Representative acceleration traces (longitudinal axis, used to calculate Fourier transforms, in bold) of flying (a) murres and (b) kittiwakes.** Fourier spectra from the same flights in **(c)** murres and **(d)** kittiwakes.

To calculate wing beat frequency, we visually selected the ten first flights of each individual from the acceleration pattern and calculated the Fast Fourier Transform in the z component, excluding the first and last 50 s of flight (to avoid changes in wing beat frequency associated with take-off and landing; see Figures 2 and 3). We considered the frequency with the strongest maximum power in the frequency domain to reflect the wing beat frequency. We also calculated partial (murres, PDBA) and overall (kittiwakes, ODBA) dynamic body acceleration at 1 s intervals, excluding the first 50 s of flight, as an estimate of energy expenditure (L1-normalized; [44],[45]). During chick-rearing, murre outgoing direction averaged 101?±?10° and incoming direction averaged 278?±?11° (N?=?38 birds; averages and standard errors generated from von Mises distribution). Consequently, we calculated wind speed in the direction of travel based on those average directions. As we focused on commuting flights, where turns were rare, we ignored energy costs associated with turning [5],[19]. We were unable to apply a recent model for mechanical flight based on pigeon flight [82] because of transient effects associated with variation in wing beat frequency.

## Results

### Ground speed and wing beat frequency

**Figure 4:**
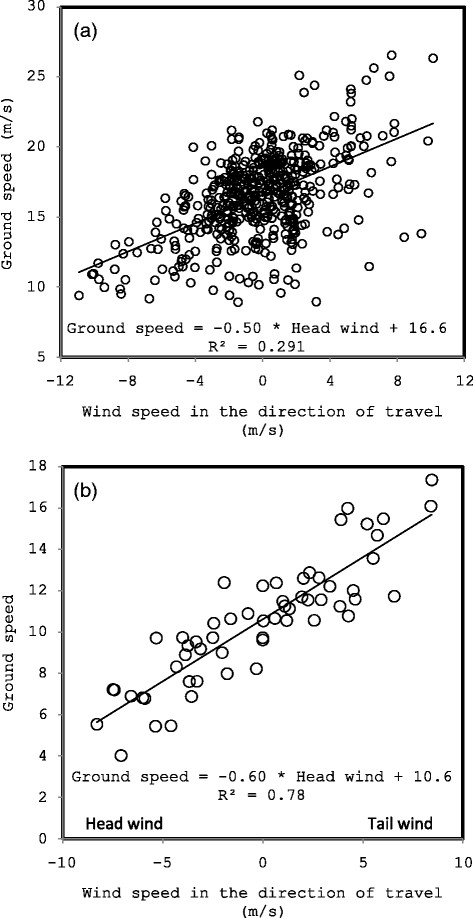
Ground speed increased with the magnitude of the component of wind speed in the direction of travel (¿tailwind¿) for both (a) murres (N = 35) and (b) kittiwakes (N = 10).

**Figure 5:**
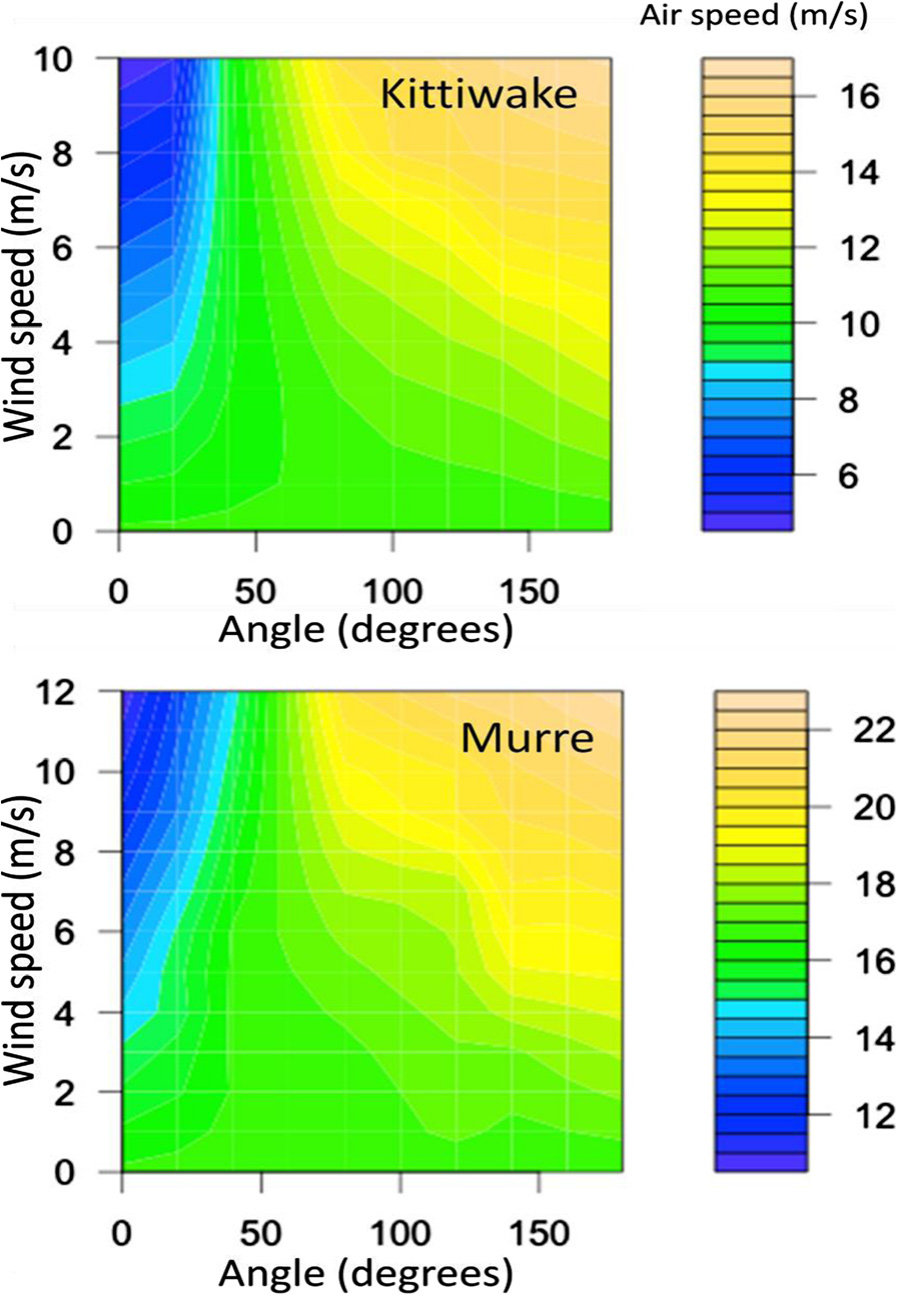
Air speed as a function of wind speed and angle between flight track and wind for kittiwakes and murres.

**Figure 6:**
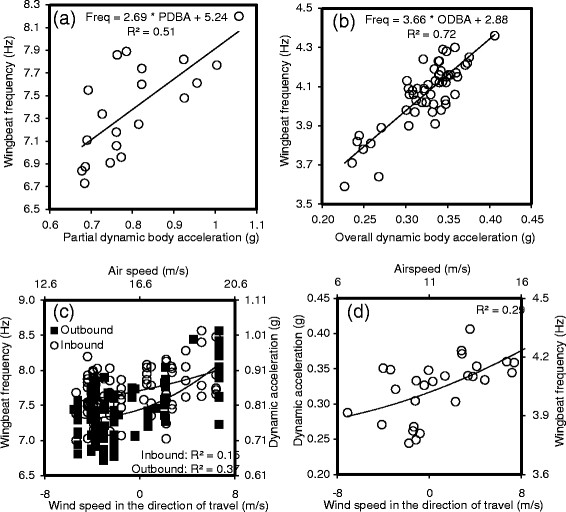
**Wing beat frequency and dynamic body acceleration were correlated for both (a) murres (N = 10) and (b) kittiwakes (N = 10).** Wing beat frequency increased with wind speed in the direction of travel for both **(c)** murres (N = 10) and **(d)** kittiwakes (N = 10). For murres, inbound and outbound wing beat frequency differed significantly, and so we show both groups. In **(c)** and **(d)**, dynamic acceleration is calculated based on the regressions in **(a)** and **(b)** and airspeed is calculated based on regressions in Figure 4.

Ground speed decreased with the magnitude of the component of wind speed against the direction of flight (Table 1, Figure 4). Crosswind speed did not affect ground speed (Table 1). In the absence of a tail or headwind, the best-fit equation generated a prediction for average ground speed of 16.6 m/s (murres) or 10.6 m/s (kittiwakes; Figure 4). Air speed was lowest with a tailwind and highest with headwind, and higher with a crosswind than without a crosswind (Figure 5). Wing beat frequency, averaged across all inbound or outbound flights for each individual, decreased with the magnitude of the component of wind speed in the direction of flight (assuming outgoing direction of 101?±?10° and an incoming direction of 278?±?11° for murres). Wing beat frequency was higher for inbound murres, carrying fish, than for outbound murres (Figure 6).

**Table 1:** Statistical output (F-values with P-values in parentheses) from general linear mixed models, with individual as a random effect, describing six foraging parameters for thick-billed murres and black-legged kittiwakes as a function of year, time of day (circularly-transformed) and windspeed

**Parameter**	**N**	**Year**	**Crosswind**	**Tailwind**	**Time**
Murres					
Outbound wing beat frequency	10	2.63 (0.11)	0.22^1^ (0.66)	***13.5***^1^ ***(0.0004)***	0.05 (0.89)
Flight speed	35	***23.2 (<0.0001)***	3.58 (0.06)	***171.1 (<0.0001)***	0.08 (0.78)
Energy delivery rate between 0600 h and 1000 h EST	82	2.61 (0.11)	**6.48 (0.01)**^2^		
Time flying per day	210	**2.96 (0.02)**	0.06 (0.81)^2^		
Residual of daily energy expenditure on body mass	49	1.53 (0.22)	2.21 (0.14)^2^		
Residual of chick growth rate on age^3^	720	**4.23 (0.04)**	0.39 (0.93)^2^		
Kittiwakes					
Wing beat frequency	10		0.11 (0.87)	**4.67 (0.04)**	0.25 (0.61)
Flight speed	10		1.58 (0.22)	***178.9 (<0.0001)***	0.01 (0.98)
Feeding rate between 900 h and 1300 h AKDT	96		**4.41 (0.04)**^2^		
Time flying per day	30		0.45 (0.51)^2^		
Residual of daily energy expenditure on body mass	37		0.01 (0.92)^2^		
Residual of chick growth rate on age^3^	126	0.49 (0.46)	3.94 (0.05)^2^		

^1^Actual direction of flight was unknown, so we assumed 101?±?10° for outbound direction (average from birds equipped with GPS).

^2^Average wind speed, rather than crosswind and tailwind components, used as independent variable. To account for the confounding effect of date on wind, average windspeed for each day was calculated as residual from the average value for a given date across all 10 years.

^3^Hatch date (murre: F_1,2876_?=?10.9 P?=?0.001; kittiwake: F_1,686_?=?2.25, P?=?0.13) was also included as a covariate.

Values in bold are statistically significant at the P?<?0.05 level and values in italics are significant at P?<?0.002.

### Behavior, diet, energy delivery rates and chick growth rates

**Figure 7:**
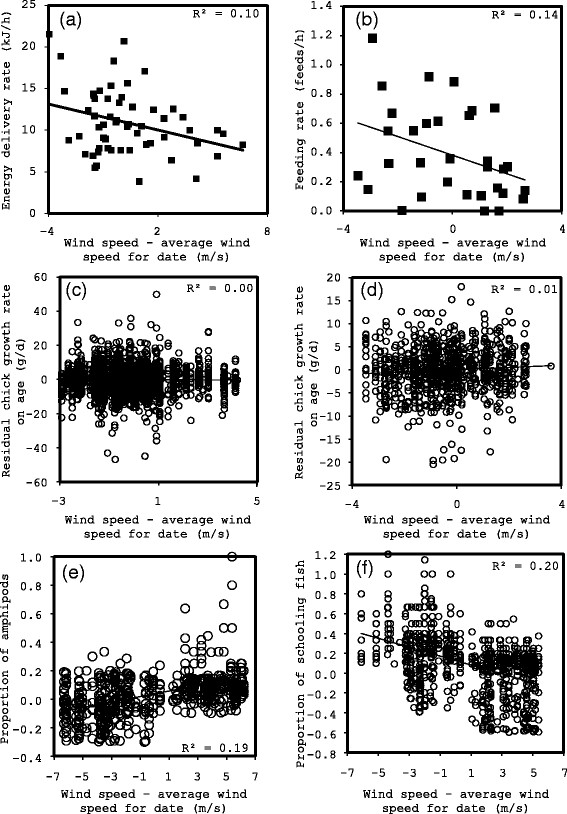
Energy delivery rate for (a) murres and (b) kittiwakes; residual chick growth rate on age for (c) murres and (d) kittiwkes; and residual proportion of (e) amphipods and (f) schooling fish, after accounting for time of day and date, relative to the difference between wind speed and average wind speed for a particular date.

Chick-provisioning rates (±SD) averaged 0.33?±?0.12 feeds h^?1^ for kittiwakes and 149?±?25 kJ d^?1^ for murres while daily energy expenditure during chick-rearing averaged 1.96?±?0.28 kJ d^?1^ g^?1^ for kittiwakes and 2.04?±?0.53 kJ d^?1^ g^?1^ for murres. Energy delivery rates decreased with wind speed (Table 1, Figure 7). Chick growth rate, daily energy expenditure and time spent flying were independent of wind speed (Table 1, Figure 7). For murres, energy delivered in the form of amphipods *Parathemisto libellula* increased with date (t_870_?=?4.00, P?<?0.0001) and headwind speed in the average direction of commuting (101?±?10°; t_870_?=??4.96, P?<?0.0001; total R^2^?=?0.26; Figure 7). In contrast, energy delivered in the form of two schooling fish, cod *Boreogadus saida* and sand lance *Ammodytes hexapterus*, decreased with date (cod: t_870_?=??2.81, P?<?0.0001; sand lance: t_870_?=?2.70, P?<?0.0001) and with headwind speed in the direction of commuting (cod: t_870_?=??4.28, P?<?0.0001; total R^2^?=?0.22; sand lance: t_870_?=??3.51, P?<?0.0001; total R^2^?=?0.18; Figure 7). Energy delivered for all other fish species was independent of wind speed.

## Discussion

At scales varying from a sub-second (wing beat frequency) to hours (feeding rates) to days (chick growth rates), the effect of wind speed was progressively buffered by seabird behavior. At the scale of seconds to minutes, we used GPS-accelerometry to demonstrate that headwinds increased instantaneous costs and birds were unable to avoid those costs, although they increased airspeed to partially compensate for reduced ground speed. At longer time scales, parents apparently buffered the effect of stormy weather on their chicks as energy delivery rates were influenced only slightly and chick growth rates were not impacted. During windy periods birds switched from unpredictable, schooling fish (sand lance and cod) to more predictable invertebrates (amphipods).

### Ground speed and wing beat frequency

Ground speed increased linearly with the component of wind speed in the direction of flight. There was no effect of crosswinds, demonstrating that birds completely compensated for crosswinds [47]-[54]. Wing beat frequency correlated with dynamic body acceleration (Figure 3a,b), as wing beat frequency is, by definition, a measure of how quickly the wing accelerates through the wing beat cycle. Variation in this relationship is likely due to variation in body mass/load, as birds carrying heavy loads will have higher wing beat frequency (work harder) but lower body acceleration (Newton¿s second law). Dynamic body acceleration is correlated with energy costs in active animals, such as seabirds [44],[45],[83],[84], so it is not surprising that wing beat frequency is a proxy for instantaneous energy costs in flight [43],[85]. For every increase in headwind of 1 m/s, ground speed decreased by only 0.5-0.6 m/s; birds increased wing beat frequency (power output) to compensate for decreasing ground speed. Thus, the increase in ground speed is not equal to the increase in wind speed and the ground speed is decreasing relative to what it would be if it were completely controlled by wind speed alone with no accommodation by the bird. The average ground speed at 0 m/s wind speed was below most past reports of murre/kittiwake ground speed (Table 2). The low flight speed may, in part, be an artifact of increased drag associated with the 25 g GPS loggers (17 g accelerometers increase murre flight costs by 21%; [12]). Indeed, it is possible that the effects throughout this paper were exacerbated due to the increased load and drag associated with instrumentation.

**Table 2:** Reported flight speeds for kittiwakes and murres

**Location**	**Technique**	**Source**	**Ground speed (m/s)**
Black-legged Kittiwake			
Middleton Island, USA	GPS logger (17 g)	Current study	10.6 (no wind)
Middleton Island, USA	GPS logger (11 g)	[68]	9.2
Varanger peninsula, Norway	Compared to car speedometer	[86]	11 (headwind: 4 m/s)
Britain	Ornithodolite	[87]	13.1
Thick-billed Murre			
Coats Island, Canada	GPS logger (25 g)	Current study	16.6 (no wind)
Coats Island, Canada	Stopwatch over known distance	[88]	20.9
Iceland	Compass data logger (29 g)	[89]	18.1
Prince Leopold Island, Canada	Stopwatch over known distance	[90]	20.1
Common Murre			
Britain	Stopwatch over known distance	[91]	22
Britain	Stopwatch over known distance	[92]	18
Britain	Ornithodolite	[87]	19.1
British Columbia, Canada	Marine radar	[93]	19.7
Sweden	GPS logger (28 g)	[94]	20.1 outbound (prevailing tailwind)
			15.1 inbound (prevailing headwind)
Russia	Stopwatch over known distance	[95]	19.4

Wind had a strong impact on the flight behavior and energy expenditure during flight of both species. The birds were able to compensate for crosswinds. In contrast, although they increased wing beat frequency in the face of increased headwinds, they were only partially able to compensate for the effect of increased headwinds.

### Buffering weather costs

Wind had a smaller impact on energy delivery rates than on flight costs and did not appear to influence offspring growth rates at all. Thus, parental birds buffered the effect of variation in wind so as not to impact their reproductive success. This is not simply because strong tailwinds on the inbound trip compensate for strong headwinds on the outbound trip; due to the nonlinear relationship between energy costs and wind speed, a return trip with 5 m/s outbound headwind and inbound tailwind imposes a 6% increase in overall costs for murres compared with the same trip with no wind. We suggest that the increased chick feeding rates during periods of high food accessibility (moderate or low winds) is due to parental murres buffering changes in food accessibility in such a way that they make up for periods of low food accessibility (high winds); parental birds may have also switched to alternative prey or sacrificed self-feeding during high winds to maintain provisioning rates.

In contrast to other studies of charadriiform seabirds [20],[60], we found no effect of wind speed on daily energy expenditure. Likewise, using a subset of the data presented here, and measuring wind speed twice a day at the colony, Elliott et al. [59] found no effect of wind on daily energy expenditure in murres. Seabirds may alter their behavior during windy days to minimize the effect of high winds; on windy days kittiwakes use formation flocks and fly at low altitudes, where wind speed is less due to the effect of the boundary layer [86]. Birds may also choose flight paths that minimize the negative effect of wind [19].

We were surprised that there was no link between several days of windy weather and reduced chick growth rates as previous studies have found measurable effects of wind on the reproductive success, attendance, feeding rates and chick growth rates of piscivorous birds [13]-[19],[23]-[29],[96],[97], contra [98]. This implies that adults of both species were adjusting their behavior to compensate for poor weather. Temporal scale likely explained some of the variation as extreme wind speeds, measurable in data collected at the scale of seconds or minutes, was not maintained for days on end. Nonetheless, there was a noticeable effect on feeding rates and flight behavior even at the reduced wind speeds (~?±?4 m/s) observed within the chick growth dataset. In support of a weather effect on above-water searching, weather in our study was unrelated to energy delivered in the form of prey items that require underwater searching but not above-water searching (e.g. benthic prey occurring well below the surface layer affected by weather). In contrast, energy delivered in the form of schooling prey that required above-water but not underwater searching (Arctic cod and sand lance) decreased on windy days. We suggest that during inclement weather (high winds), birds were unable to track schooling fish due to high wave height or difficulty flying, and switched to more abundant, but lower energy content [38], amphipods and benthic fish. The effect of date was also apparent for several of these parameters as murres deplete food sources over the course of the season and switch to alternative, less profitable food sources [63].

If parental birds have the capacity to increase feeding rates to offspring during poor weather, why then do they not do so during good weather? One possibility is that they use periods of good weather to replenish their own reserves, which are larger and less likely to be exhausted during poor weather. Furthermore, the gain to the parent in terms of increased probability of chick survival may be a decelerating function of energy delivery rates; a chick that starves represents zero reproductive success but a very heavy chick may actually have low survival if it has difficulty fledging [99],[100]. Likewise, both the chick and adult are only able to assimilate a certain amount of food each day [12], and so there is no reason to catch excess food. Finally, during periods of low food the parent may divert more energy towards the chick simply in response to increased begging, and that response may not be linearly tied to ultimate costs and benefits [100]-[102].

## Conclusions

Windscapes alter the prey field accessible to marine predators by altering their ability to locate schooling prey [19],[103],[104]. Thus, wind acted as a dimension within the N-dimensional animal energetic niche [105],[106]. Marine predators¿murres and kittiwakes¿altered their behavior as their energetic niche varied, showing flexibility in their behavioral response to the variable marine environment [39],[40],[106],[107]. Such flexibility proves key to maintaining fitness across variable environmental conditions.

## Competing interests

The authors declare that they have no competing interests.

## Authors¿ contribution

All co-authors participated in the writing of the manuscript. AG, KE, LC and OO collected the field data. AK, KE and LB collated and analyzed the data. JS supervised the doubly-labelled water analyses. AK and YRC customized and supplied the LUL, Little Leonardo and GPS units. All authors read and approved the final manuscript.
